# The Influence of Nurse Education Level on Hospital Readmissions—A Cost-Effectiveness Analysis

**DOI:** 10.3390/ijerph19074177

**Published:** 2022-03-31

**Authors:** Beata Wieczorek-Wójcik, Aleksandra Gaworska-Krzemińska, Aleksander Owczarek, Michał Wójcik, Monika Orzechowska, Dorota Kilańska

**Affiliations:** 1 Department of Nursing and Medical Rescue, Pomeranian University in Slupsk, 76-200 Slupsk, Poland; beata.wieczorek-wojcik@apsl.edu.pl; 2 Institute of Nursing and Midwifery, Medical University of Gdansk, 80-210 Gdansk, Poland; 3 Health Promotion and Obesity Management Unit, Department of Pathophysiology, Faculty of Medical Sciences in Katowice, Medical University of Silesia in Katowice, 40-055 Katowice, Poland; aowczarek@sum.edu.pl; 4 Rehazentrum Walenstadtberg, Chnoblisbüel 1, CH-8881 Walenstadtberg, Switzerland; michal.wojcik@kliniken-valens.ch (M.W.); monika.orzechowska@kliniken-valens.ch (M.O.); 5 Department of Coordinated Care, Medical University of Lodz, 90-419 Lodz, Poland; dorota.kilanska@umed.lodz.pl

**Keywords:** cost-effectiveness analysis, education level, nurse staffing, readmissions, missed care

## Abstract

Background: Readmissions are adverse, costly, and potentially preventable. The study aimed to evaluate the cost-effectiveness of reducing readmissions resulting from missed care, depending on the level of education of nurses, from the perspective of the service provider. Methods: We calculated missed care resulting in additional readmissions based on the longitudinal study conducted between 2012 and 2014, as well as readmissions that could have been potentially prevented by adding a 10% increase in hours of nursing care provided by BSN/MSc nurses for 2014. The cost-effectiveness analysis (CEA) was performed to calculate the cost-effectiveness of preventing one hospitalization in non-surgical and surgical wards by increasing the number of nursing hours provided by BSN/MSc nurses. Cost–benefit analysis (CBA) was performed, and the CBR (cost–benefit ratio) and BCR (benefit–cost ratio) were calculated. Results: Increasing the number of hours of nursing care (RN) by 10% decreased the chance for an unplanned readmission by 11%; (OR = 0.89; 95% CI: 0.78−1.01; *p* = 0.08) in non-surgical wards and 43% (OR = 0.57; 95% CI: 0.49−0.67; *p* < 0.001) in surgical wards. In non-surgical wards, the number of readmissions that were preventable with extra hours provided by BSN/MSc nurses was 52, and the cost-effectiveness ratio (CER) was USD 226.1. The number of preventable readmissions in surgical wards was 172, and the CER was USD 54.96. In non-surgical wards, the CBR was USD 0.07, while the BCR was USD 1.4. In surgical wards, the CBR was USD 0.02, and the BCR was USD 4.4. Conclusions: The results of these studies broaden the understanding of the relationship among nursing education, patient readmission, and the economic outcomes of hospital care. According to the authors, the proposed intervention has an economic justification. Hence, the authors recommend it for approval by the service provider.

## 1. Introduction

Readmissions are disturbingly, occurring in one of five hospitalized patients [1,2]. In Poland, the readmission rate for patients readmitted to the same hospital and the same ward type as initial hospitalization is 19.2%. The overall ratio for 90 days is over 30% [3]. Readmission is defined as an unplanned admission to the hospital within 30 days from the previous discharge, unless the previous discharge indicated the need for readmission [4,5]. The lack of continuity of care between in-hospital and post-hospital care is one of the main risk factors of readmissions [1,6]. During hospitalization, patients often receive new medications, their therapeutic regimens are changed, and they do not receive effective guidance. A high percentage of patients readmitted shortly after discharge to the same hospital may indicate insufficient in-hospital care quality or inadequate care coordination after discharge [1]. The results of previous studies indicate that for more than 30% of patients, discharge recommendations are not followed because outpatient doctors and nurses are not familiar with the recommendations given to the patient at discharge [7].

Preventing readmission is a priority of health policy in many countries. For example, in the USA, the federal government’s campaign to reduce the frequency of readmissions by applying financial disincentives has entered its 10th year of implementation in Medicare. Payments to 2499 hospitals will be lowered throughout the current fiscal year through September 2022. The fines can be considerable; they reached up to USD 217,000 on average per hospital in 2018. Medicare estimates that the penalties over the next fiscal year will save the government USD 521 million. Hospital readmissions have become less frequent since the Affordable Care Act (ACA) was enacted, primarily due to the financial threat of the penalties [8,9].

The studies by Feigenbaum et al. indicate that almost half of the 30-day readmissions can be prevented, and 11% of total readmissions can be prevented entirely [5]. When readmissions are prevented, the hospital accrues savings from the non-reimbursed cases, whereas savings from reimbursed readmissions are received by public and private payers [10].

Hospitals are pursuing a variety of strategies to reduce readmissions. One of the crucial actions is to improve care coordination and communication between care providers and patients and patient education [11,12,13,14,15,16].

In recent years, there has been an increase in publications emphasizing the importance of advanced nursing practice and nursing education on improving patient outcomes and reducing the incidence of adverse events, including readmissions [17,18,19,20,21,22,23]. Bryant-Lukosius et al. found that in the case of elderly patients and caregivers, CNS (clinical nurse specialist) care improved caregiver depression and reduced frequency and length of stay and readmission costs [24]. As Audet et al. observed, “while evidence suggests that higher nurse education is associated with lower risks of mortality and failure to rescue, longitudinal studies are needed to better ascertain these associations and determine the specific thresholds that minimize risks” [25].

Lasater et al. proved that patients in hospitals that increased their proportion of BSN (bachelor nursing degree) nurses over time had significantly reduced odds of risk-adjusted mortality, 7-day readmission, and 30-day readmission, as well as shorter lengths of stay. Longitudinal findings of an association between increased proportions of BSN nurses and improvements in patient outcomes corroborate previous cross-sectional research, suggesting that a better-educated nurse workforce may add value to hospitals and improve patient outcomes [26].

In Poland, re-hospitalization research is very limited. Usually, studies focus on clinical and social aspects. Research on CEA has never focused on the level of education of nurses from the perspective of the service provider [27].

However, it should be remembered that the authors of most studies emphasize that the results should be treated with caution due to the limited quantity and quality of published studies and insufficient reliability of some evidence; hence, further research is necessary. More than ten years ago, the report of the Institute of Medicine (IOM), “The Future of Nursing: Leading Change, Advancing Health”, called for increasing the proportion of BSN nurses in the workforce to 80% by 2020 [28].

The validity of this recommendation was confirmed by Yakushev et al., who found that a continuous BSN proportion was associated with lower mortality. Compared with patients with less than 80% BSN care, patients receiving more than 80% of care from BSN nurses had lower odds of readmission and a 1.9% shorter length of stay. Moreover, economic simulations support a strong business case for increasing the proportion of BSN-educated nurses to 80% [29]. Additionally, the review of studies by O’Brien et al. confirms that mortality, failure to rescue, and readmission rates all decrease as the proportion of BSN nurses increases in the nursing staffing ratio [30].

Despite these recommendations and research results, Aiken observes that it is challenging to find institutions in the US with 80% of BSN/MSc nurses [17]. Hence, robust economic evaluations are still needed to address cost-effectiveness.

Since 2002, there has been only one nursing education pathway in Poland: obtaining a bachelor’s degree as per the EU directive, i.e., completing a program covering six semesters and 4600 h of study [31]. Currently, approximately 30% of nurses in Poland have higher education [32]. Unfortunately, due to the considerable shortages of nurses in the healthcare system, politicians or local authorities sometimes postulate returning to the previous model of education, which did not require taking the final high school examination and was a shorter path to the labor market. Hence, research that provides scientific evidence about the validity of higher education of nurses is fundamental. Based on the research, it is possible to indicate significant relationships between the level of nursing education and care outcomes and indicate economic benefits for the healthcare system. From the point of view of the employer and payer, economic benefits are crucial, so such analyses are necessary, however scarce.

This study aims to assess the cost-effectiveness of eliminating readmissions as a missed care effect, depending on nurses’ level of education, from the perspective of the service provider.

## 2. Methods

A retrospective longitudinal observational study (2012–2014) explored readmissions concerning nursing education level.

### 2.1. Population

The study included patients from 8 wards, including 4 non-surgical and 4 surgical wards. In the analyzed period, the study population consisted of 44,809 patients. The cost-effectiveness analysis included a population of 14,369 patients from 2014 (61,536 patient days). This included 6031 patients hospitalized in non-surgical wards (43,502 patient days) and 8987 patients in surgical wards (28,063 patient days). The number of patient days is the number of hospitalized patients multiplied by the number of days spent in the hospital.

### 2.2. Assessment of the Education Level of Nurses

The study covered all nurses working in the wards, a total of 528 positions. Nurses’ data were collected by a proprietary tool developed based on the MS Excel program. The calculations were completed with a one-day frequency. In this study, a nurse was defined as a nursing school graduate who, depending on the period of education, had completed vocational studies and obtained a bachelor’s (BSN) and master’s degree (MSc) in nursing. In all cases, it was a general nurse. The number of nurses was monitored for each shift. The ward nurses supplemented the data. The percentage of BSN/MSc nurses was calculated based on the collected data. Then, a comparison was performed between the working hours of BSN/MSc nurses and nurses without higher education. Work shifts and number of work hours constituted the base for the nurses’ wages. The calculations were performed quarterly for each year of the study. The cost of one hour of care provided by a nurse with higher education was calculated by dividing the total salary of BSN/MSc nurses by the actual number of hours of care provided by BSN/MSc nurses. The same principle was used to calculate the costs of one hour of care by a nurse without higher education. There were separate calculations for surgical and non-surgical wards.

### 2.3. Readmissions Assessment

The follow-up study covered three consecutive years, from January 2012 to December 2014. The analyzed period covered 12 quarters in total. For the studied period, readmissions were calculated for 1000 patient days in non-surgical wards: general, pulmonology, neurology, and cardiology, and surgical wards: general surgery, urology, laryngology, and orthopedics. Readmission was defined as an unplanned admission to the hospital within 30 days from the previous discharge unless the previous hospitalization was concluded with an indication for readmission [4]. Data on readmissions were collected in digital patient records and maintained in EPR (Electronic Patient Record): CLININET and SERRUM systems [33]. In 2012, the CLININET digital record system was implemented at the hospital, replacing the previously used SERRUM. Transferring all data from the SERRUM to the CLININET program was a critical challenge. Therefore, the study included data for 2014 that met the criteria for performing the calculations, and data from the period 2012–2013 were excluded.

### 2.4. Cost and Cost-Effectiveness Evaluation

The cost-effectiveness analysis (CEA) method used in the study was based on the readmissions ratio calculated for 1000 patient days. The last year of the study (2014) was included in the cost-effectiveness analysis because the costs of services from that period are closer to the current costs. In addition, until the change in the IT system in 2014, the data resource was suboptimal and could not guarantee a reliable analysis. The CEA analysis assumed that patients would receive additional hours of care from BSN/MSc nurses in both non-surgical and surgical wards. The cost of an hour of work of a nurse with higher education was calculated according to Cooxon methodology by subtracting the cost of working hours of all nurses in non-surgical and surgical wards, irrespective of the level of education. The adopted variable used for comparison was the number of hours of BSN/MSc nurses resulting from the current work schedule of the hospital. The cost of preventing one readmission was adopted to measure the cost-effectiveness analysis results—(CER—cost-effectiveness ratio). The cost of the effect unit was compared to the revenues achieved by the service provider per patient. The next step was calculating the number of readmissions that could have been prevented by increasing, by 10%, the number of hours of BSN/MSc nurses—it was performed by dividing the cost of additional hours of BSN/MSc nurses by the preventable readmissions ratio per 1000 patient days.

The cost-effect ratio (CER) was calculated by dividing the cost of additional hours of BSN/MSc nurses working in non-surgical and surgical wards by the number of preventable readmissions. In the multivariate analysis, the education of nurses proved to significantly influence the number of preventable readmissions, as opposed to the number of nurses per patient per day (NHPPD). The difference in the readmissions coefficient between nurses’ care depending on the level of education (incremental change) was calculated.

CBA (cost–benefit analysis) was used to verify the cost-effectiveness of the increase in the number of hours of BSN/MSc nurses. The gross monetary benefit was calculated for healthcare providers by multiplying per patient revenue for the payer (NFZ—Polish National Health Fund) by the total number of preventable readmissions in non-surgical and surgical wards. We calculated how much has to be spent in PLN (Polish currency; PLN 1 ≈ USD 3.507) to save PLN 1. This analysis was performed using the CBR (cost–benefit ratio) and the BCR (benefit–cost ratio). The result obtained in PLN was converted into USD.

The study was approved by the Bioethics Committee of the Medical University of Gdansk under no. NKBBN/41/2015.

## 3. Results

In 2012–2014, there were 2029 readmissions. The readmission rate was 9.77 per 1000 patient days. Due to the lack of data, the readmissions rate was not calculated for the first three quarters of 2012. In the fourth quarter of 2012, it was 8.19 per 1000 patient days and was lower than in the subsequently analyzed quarters.

**Figure 1:**
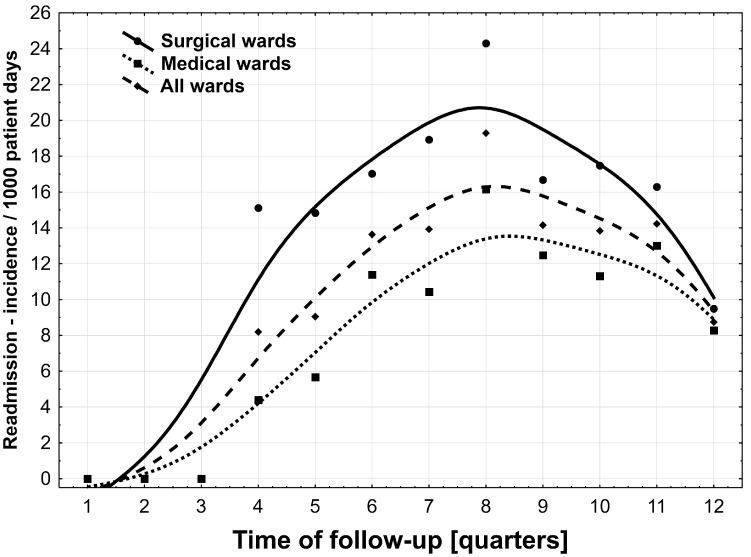
Readmission rate in the following quartiles in 2012–2014, in total as well as in surgical and non-surgical wards.

For the mean readmission rate (per 1000 patient days), no statistically significant difference was found between surgical and non-surgical wards in the study. This applied to all 12 analyzed quarters (12.51 ± 8.24 vs. 7.76 ± 5.64; *p* = 0.11). Figure 1 and Table 1 present the readmission rates for the hospital, surgical, and non-surgical wards in the analyzed period.

In 2014, 910 readmissions were recorded, including 490 (53.8%) in non-surgical and 420 (46.2%) in surgical wards. Readmissions to non-surgical wards included: 274 (55.9%) to general wards, 57 (11.6%) to pulmonology, 132 (27.0%) to cardiology, and 27 (5.5%) to neurology. In surgical wards, readmission included: 181 (43.1%) to general surgery, 39 (9.3%) to orthopedics, 170 (40.5%) to urology, and 30 (7.1%) to laryngology.

In 2014, 177,723 h of nursing care were provided in non-surgical wards, including 59,027 h by BSN/MSc nurses. In surgical wards, the numbers were 150,969 h and 69,595 h, respectively.

In the analyzed non-surgical wards, the hours of work provided by BSN/MSc nurses accounted for 32.2%, and in surgical wards, they comprised 42.3%. A cause–effect relationship was demonstrated between the number of nursing hours provided by BSN/MSc nurses and readmission. The more nursing hours provided by BSN/MSc nurses, the fewer readmissions in non-surgical and surgical wards. An increase in the nurses’ rate by 10% in those wards caused a decrease in readmissions by 8.8 per 1000 patient days and by 24.7 per 1000 patient days, respectively. Backward stepwise regression analysis (β (SE)/100) indicated an impact of nurses’ education on the reduction in readmissions both in non-surgical wards (−0.879; standard deviation SD = 0.283) * and in surgical wards (−2.474; SD = 0.700) ^#^ (* *p* < 0.05, ^#^ *p* < 0.01).

An increase in the percentage of nurses (RN) from 32.2% to 42.2% in non-surgical wards decreased the chance of an unplanned readmission by 11%; however, this dependence was statistically insignificant (OR = 0.89; 95% CI: 0.78 − 1.01; *p* = 0.08). An increase in the percentage of professional nurses from 42.3% to 52.3% in surgical wards decreased the risk of an unplanned readmission by 43% (OR = 0.57; 95% CI: 0.49−0.67; *p* < 0.001).

### 3.1. Cost-Effectiveness Analysis (CEA)

The received costs in PLN were converted into USD according to the conversion rate for the date of calculations (3.507). The total salary of nurses employed in non-surgical wards was USD 799,345.4, including the salary of BSN/MSc nurses, which was USD 388,438.7. In the surgical wards, the salary rates were USD 804,439.0 and USD 465,461.8, respectively. The average cost of one nursing hour in a non-surgical ward was USD 4.43, and the cost of one nursing hour from a graduate nurse was USD 6.55 on average. For the surgical wards, it was USD 5.33 and USD 6.69, respectively. The average revenue per patient was calculated by dividing the contract value by the number of hospitalized patients in a given period. In the analyzed year, the average revenue was USD 1116.42 in non-surgical wards and USD 841.53 in surgical wards (Table 2).

The number of additional nursing hours provided by BSN/MSc nurses in non-surgical wards was 5905.2, while the total cost of these hours was USD 11,730.62. In surgical wards, the number of additional hours of BSN/MSc nurses was 6953.4, and the cost of these hours of BSN/MSc nurses was USD 9462.6. The calculation indicates that in non-surgical wards, the number of readmissions that are preventable through additional hours of BSN/MSc nurses was 52, and the CER, i.e., the cost of preventing one readmission (with a 10% increase in the percentage of hours of BSN/MSc nurses) was USD 226.09. In surgical wards, the number of readmissions that were preventable through additional hours of BSN/MSc nurses was 172, and the CER was USD 54.96 (Table 3).

The results indicate that an increase of 10% of BSN/MSc nurses resulted in an 11% lower chance of readmissions. The patients of surgical wards were 43% less likely to be readmitted.

Thanks to the additional working hours of BSN/MSc nurses in non-surgical wards, readmissions decreased from 11.3 to 10.1 per 1000 patient days, which is a 1.20 incremental change per 1000 patient days. In surgical wards, in turn, the readmission rate was reduced by nearly half: from 15.0 to 8.8. The incremental change was 6.15 per 1000 patient days.

### 3.2. Sensitivity Analysis

A deterministic multidirectional sensitivity analysis was performed. It included variables with high uncertainty/variability in the analyzed range (Table 4). After simulating the optimistic and pessimistic scenarios, objective results were obtained reflecting the possible effects of a 10% increase in the nursing hours provided by BSN/MSc nurses in non-surgical and surgical wards. The aim of this analysis was to estimate to what extent the planned intervention was at risk of yielding a result inconsistent with the forecast in the economic analysis when affected by the change in the number of hours of BSN/MSc nurses, the cost of working hours of BSN/MSc nurses, and the number of preventable readmissions.

Table 4 presents the parameter values used as the base cases for the sensitivity analysis and lower and upper values.

Multilevel analysis of sensitivity was conducted. Variables included the level of certainty in the following areas: the number of nursing hours provided by BSN/MSc nurses, the cost of one nursing hour provided by a BSN/MSc nurse, and the number of preventable readmissions (Table 5).

For the variable of the number of nursing hours provided by BSN/MSc nurses in non-surgical and surgical wards, the standard deviation was calculated from the quarterly data for nursing hours (base number of hours) for 2014. The standard deviation hours were then subtracted from the BSN/MSc number (pessimistic scenario) or added to the base number of hours (optimistic scenario). The calculated standard deviation was 17% for the non-surgical wards for the non-surgical wards and 22% for the surgical wards.

For the variable of the cost of one hour provided by a BSN/MSc nurse, the deviation was calculated from the quarterly data of non-surgical wards from 2014. The deviation cost was added to the cost of an hour provided by a BSN/MSc nurse (pessimistic scenario) or deducted from the cost of an hour provided by a BSN/MSc nurse (optimistic scenario). The standard deviation value was 21% for non-surgical and surgical wards.

For the preventable readmissions variable, we adopted the base readmission coefficient for 2014. Then, the lowest readmission coefficient was selected from the four quarters, and the number of preventable readmissions was calculated (pessimistic scenario). This ratio was 8.3% in non-surgical wards and 9.5% in surgical wards. Then, the highest readmission coefficient was selected from the four quarters, and the number of preventable readmissions was calculated (optimistic scenario). This ratio was 17.5% in non-surgical wards and 13% in surgical wards.

In the sensitivity analysis performed, reducing the number of nursing hours to 4899.2 in non-surgical wards would reduce the CER to USD 200.3 (optimistic scenario). In the pessimistic scenario, increasing the number of hours to 6906.2 h would increase the CER to USD 282.6. In the optimistic scenario, if the number of preventable readmissions was increased by the standard deviation to 60 readmissions, the CER would decrease to USD 176.2. In the pessimistic scenario, with the readmission number dropping to 38, the CER would reach USD 269.3.

In surgical wards, reducing the number of nursing hours to 5428.39 would lead to the CER dropping to PLN 42.87 (optimistic scenario). In the pessimistic scenario, an increase in the number of hours to 8490.57 h would increase the CER to USD 67.05. If the number of preventable readmissions was increased to 201, the CER would drop to USD 52.09. In the pessimistic scenario, if the number of readmissions dropped to 109, the CER would increase to USD 56.37. If the cost of an hour provided by a BSN/MSc nurse was increased by the standard deviation, the CER would reach USD 111.73. The optimistic scenario does not assume a decrease in the cost of an hour of care, so the variable has no impact on the CER.

The difference in the CER between surgical and non-surgical wards is notable. The higher baseline CER rate in non-surgical wards results mainly from fewer preventable readmissions (8.8 for non-surgical wards and 24.7 per 1000 patient days for surgical wards).

**Figure 2:**
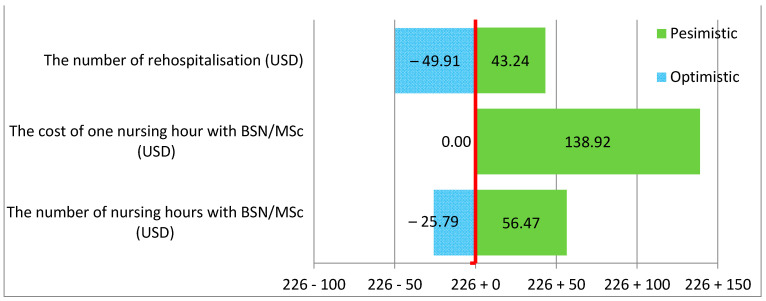
Increasing the percentage of care provided by nurses with BSN/MSc degrees by 10% in non-surgical wards.

**Figure 3:**
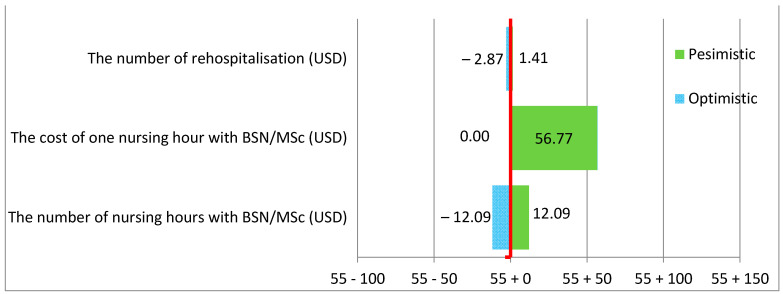
Increasing the percentage of care provided by nurses with BSN/MSc degrees by 10% in surgical wards.

As indicated by the sensitivity analysis, the greatest threat to the non-surgical and surgical wards results from the change in the cost of one hour of a BSN/MSc nurse. The number of additional hours of BSN/MSc nurses was lower in non-surgical wards, but the cost of one nursing hour was higher, which resulted in a higher total cost compared to surgical wards. The results of the variables are presented in the tornado diagram below (Figure 2 and Figure 3).

As indicated in the tornado diagram, there was a much greater risk of an increase in the cost of preventing one readmission (CER) in non-surgical than surgical wards.

The base CER in non-surgical wards was 226.09 and was much higher than in surgical wards at 54.96. The variable that was the most significant risk factor and may have the most negative impact on the cost of preventing one readmission, and thus on the hospital’s budget, was the cost of one hour of work of a nurse with higher education. In non-surgical wards, the change in the cost of one hour of a BSN/MSc nurse increased the cost of preventing one readmission by USD 138.92; in surgical wards, this increased the cost by USD 56.77. At the same time, it can be seen that the change in the number of additional BSN/MSc nursing hours also had a considerable impact on the value of the CER. The change in the number of extra hours of BSN/MSc nurses resulted in a CER increase of USD 56.27 in non-surgical wards and USD 12.09 in surgical wards.

CBA (cost–benefit analysis) showed a gross monetary benefit for the healthcare provider of USD 46,323 in non-surgical wards and USD 135,430.21 in surgical wards. In 2014, the revenue per patient was USD 1116.42 in the non-surgical wards and USD 841.53 in the surgical wards. We calculated how much has to be spent in PLN to save one PLN. The CBR (cost–benefit ratio) indicates that each zloty saved by non-surgical wards by increasing the number of BSN/MSc nursing hours cost USD 0.07 (PLN 0.25). Additionally, the BCR (benefit–cost ratio) was USD 1.41 (PLN 5), which means that every zloty invested in increasing the number of BSN/MSc nursing hours yielded the benefit of USD 1.41 (PLN 5). In surgical wards, the CBR (cost–benefit ratio) indicates that each zloty saved by increasing the number of BSN/MSc nursing hours cost USD 0.02 (PLN 0.07). Additionally, the BCR (benefit–cost ratio) was USD 4.37 (PLN 15), which means that every zloty invested in increasing the number of BSN/MSc nursing hours yielded the benefit of USD 4.37 (PLN 15).

## 4. Discussion

Data available in the United Kingdom and the United States indicate that one in five patients is readmitted within 30 days of discharge [34]. In hospitals in the USA, approximately 6.8% of patients are treated in emergency wards within seven days of the previous discharge, and as many as 31% are readmitted [35]. Depending on the study, the readmission rates in the United States are 8.5–21.9% [36], 5–29% [2], or 13.8–21.8% (2008) [35]. The readmission rate per 1000 patient days is also discussed. The NQF Report of 2012 indicates that the readmissions ratio per 1000 patient days is at the level of 4.4 [37]. The study by Lynn from 2014 found that the readmission coefficient was 15.1 [38]. In another study from this period, the rate was 6.8 per 1000 patient days [39]. Readmission rates vary across countries and geographies, depending on the rates of chronic conditions and the intensity of treatment in a given hospital [35].

It should be emphasized that readmissions within 30 days of hospitalization are unfavorable, costly, and potentially preventable [40]. Unplanned readmissions within 30 days of discharge can be effectively prevented, even though the reasons for readmissions are multifactorial and potentially associated with comorbidities [7,41,42].

The working conditions of nurses also affect readmissions. A positive work environment, including the level of nursing staffing, has been shown to affect the rate of 30-day readmissions [43]. Hence, improving the working environment conditions by creating a positive practice environment is conducive to reducing readmission.

Available research also points to health literacy issues. Failure to prepare the patient or their caregivers for discharge also contributes to readmissions [7,44]. It has been proven that the readmission rate can be reduced by 5% or more if the quality of the patient’s preparation for discharge improves [35]. Additionally, the patients and their caregivers often indicate a lack of adequate preparation for discharge, and their needs for access to information remain unmet [45,46,47,48]. Evidence indicates that educational activities are undertaken by qualified personnel, i.e., educating patients and their families about care and support, reduce readmissions within 30 days after discharge. Nurses play a crucial role in discharge planning, coordinating care, and education [49].

The quality of patient education provided by nurses before discharge should also be emphasized, as it is related to patient readiness for hospital discharge [50] and addresses inadequate health literacy. Introducing solutions such as education through telenursing at the hospital level can be beneficial to prevent readmissions, especially in the current pandemic situation. In the current benefit refund system, the payer covers the costs of the telenursing services within the primary healthcare system. The same benefit could be financed at the hospital level and could further reduce readmissions, as demonstrated for heart failure cases [51,52]. Access to modern technologies such as telemonitoring has been proven to influence readmissions of high-risk patients and can reduce the readmission rate by up to 15% [42].

The care model also influences readmissions. Promising results were achieved in reducing readmissions in the case of changing the care model through coordination [53].

It should be emphasized that none of these studies were carried out in Poland. Even though some of them yielded statistically significant results, it should be remembered that they came from various countries with specific health policies.

This is the first study in Poland analyzing the cost-effectiveness of eliminating readmissions as a missed care effect by employing BSN/MSc nurses. The results indicate that an increase of 10% in BSN/MSc nurses result in an 11% lower chance of rehospitalization. The patients of surgical wards are 43% less likely to be readmitted. The cost of preventing one readmission (CER) by increasing BSN/MSc nurse employment by 10% was USD 226.09 in non-surgical wards and USD 54.96 in surgical wards.

In the conducted economic analysis of CEA, based on the population of adult patients of non-surgical and surgical wards, the cost-effectiveness assessment covered an intervention consisting of increasing the number of working hours of BSN/MSc nurses by 10%. This was related to the current number of hours of BSN/MSc nurses, which is 32.2% in non-surgical and 42.3% in surgical wards. It was assumed that the clinical outcome would be reducing the number of readmissions. The profitability analysis was carried out from the perspective of the provider, i.e., the hospital. In research conducted by Yakusheva et al., economic simulations for a single hospital indicated that an increase in BSNs to 80% for each patient could potentially mean USD 5.6 million in savings per year, mainly due to a lower number of readmissions and slightly shorter stays, which would overcompensate for the annual costs of about USD 1.8 million in increased salaries related to BSN qualifications [29]. Our study results indicate that the costs of the described intervention, consisting in increasing the number of hours by BSN/MSc nurses by 10%, are high, especially for non-surgical wards. However, the effects of preventing 224 readmissions in total in both types of wards are also high. The analysis carried out for the publication indicates that the examined intervention belongs to the category of more expensive and more effective interventions [51]. According to the HB HTA guidelines in Poland, interventions in this area are currently not considered for reimbursement by the payer. Hence, this cost becomes the cost of the provider [54]. The healthcare provider needs to establish the maximum bearable cost of one hour of nursing care, including BSN/MSc nurses and an acceptable CER threshold.

The CEA provides evidence that the intervention is not economically attractive to the provider. However, employing additional BSN/MSC nurses may lead to reimbursement for the hospital for the funds spent on treating readmitted patients. In the case of readmissions, the hospital covers the costs of treating the patient, including possible complications and hospital infections. Instead, the funds could be used to cover the additional hours of BSN/MSc nurses. Therefore, a reduction in the number of readmissions would offset the increased labor costs due to the increase in the cost of BSN/MSC nursing hours.

In the Polish insurance system, the payer refunds readmissions over 14 days after discharge. The healthcare provider bears the costs of readmissions up to 14 days after discharge, while the payer (National Health Fund) covers those occurring over 14 days after discharge. The calculated net monetary value is USD 46,323 for the non-surgical wards and USD 135,430.21 for the surgical wards, assuming that the hospital receives funding for preventable readmissions. From the CBA perspective, there is beneficial cost-effectiveness. Even with a refund of half of the readmissions, i.e., 15 to 30 days after discharge, the intervention is still cost-effective. According to Aiken, the results of the study by Yakusheva et al. on the business justification for increasing the number of BSN nurses should catalyze the transformation that has been going on for a long time and remains in the public interest [16].

The results of the CEA and CBA analyses are intended to support the decision-making process of hospital boards. Even the most cost-effective interventions can be beyond the reach of the hospital budget and vice versa; cost-effective interventions can be beneficial from the perspective of quality of care. Therefore, the decision of the Hospital Management Board to accept additional working hours of BSN/MSC nurses requires information on the impact of this intervention on the budget of the healthcare provider and the costs of adverse events. The tornado diagram indicates which variables the healthcare provider should consider when deciding to implement an intervention.

The national and international HTA guidelines recommend that a budget impact analysis (BIA) [55] should be carried out in addition to economic analyses.

## 5. Conclusions

The research into the business case for the investment in employing BSN/MSc nurses yields results confirming the viability of such changes and their concordance with public interest. It transpired that implementing the proposed indicator to prepare the budget for nursing care and reduce the number of readmissions at the hospital level as part of health policy is crucial to reducing the costs of readmissions and increasing the availability of other patients’ services.

### Limitations

The study included patients and nurses from one medical entity. To obtain a wider range of data, it is necessary to extend the scope of the study to other medical entities in accordance with the HTA methodology. The study did not take into account the social perspective and the payer, but only the perspective of the service provider, i.e., the hospital. The assumptions adopted for the sensitivity analysis concerning the change in the cost per hour of care provided by nurses with higher education were considered the most probable. However, the situation of the labor market and the growing expectations of employees may force a greater change in the hourly cost of care, including for nurses with higher education.

In 2017, the Primary Healthcare Act was introduced in Poland. Some of its regulations regarding coordinated healthcare came into force in October 2021, while others are scheduled be put into practice in 2025. This change may result in better post-hospital care in the future and, consequently, may reduce the number of hospitalizations.

## Figures and Tables

**Table 1:** Readmission rate in all wards as well as in non-surgical and surgical wards in 2012–2014.

Hospital		2012				2013				2014				
Ward	N	I	II	III	IV	I	II	III	IV	I	II	III	IV	Total
Surgical wards	Readmissions	N/A *	N/A *	N/A *	89	109	142	156	164	123	124	106	67	1080
	Patient days	6630	6009	5759	5891	7350	8344	8246	6750	7373	7095	6509	7057	83,013
	Readmissions/1000 patient days	N/A *	N/A *	N/A *	15.1	14.8	17.0	18.9	24.3	16.7	17.5	16.3	9.5	13.0
Non-surgical wards	Readmissions	N/A *	N/A *	N/A *	47	71	144	123	174	139	116	143	92	1049
	Patient days	11,534	11,142	10,163	10,714	12,535	12,642	11,794	10,767	11,135	10,255	10,995	11,117	134,793
	Readmissions/1000 patient days	N/A *	N/A *	N/A *	4.4	5.7	11.4	10.4	16.5	12.5	11.3	13.0	8.3	7.8
Total	Readmissions	N/A *	N/A *	N/A *	136	180	286	279	338	262	240	249	159	2129
	Patient days	18,164	17,151	15,922	16,605	19,885	20,986	20,040	17,517	18,508	17,350	17,504	18,174	217,806
	Readmissions/1000 patient days	N/A*	N/A*	N/A*	8.2	9.1	13.6	13.9	19.3	14.2	13.8	14.2	8.8	9.8

* no data due to transfer issues between the SERRUM and the CLININET.

**Table 2:** Costs and revenue and number of nursing hours, including BSN/MSc nurses in non-surgical and surgical wards.

Wards	Gross Salary of Nurses (USD)	Gross Salary of BSN/MSc Nurses (USD)	Total Number of Nursing Hours	Number of Nursing Hours Provided by BSN/MSc Nurses	Revenue per Patient (USD)
Pulmonology	119,101.80	41,946.96	32,487.00	6237.50	965.80
General Ward	267,606.50	175,016.25	57,330.00	22,014.72	1194.22
Neurology	201,972.63	99,507.27	42,042.00	19,675.66	1513.09
Cardiology	210,664.50	71,968.18	45,864.00	11,099.09	894.86
Non-surgical wards TOTAL	799,345.42	388,438.67	177,723.00	59,026.97	1116.42
General surgery	330,448.25	232,045.05	66,885.00	36,786.75	896.78
Urology	169,470.43	96,665.98	26,754.00	13,430.51	595.95
Laryngology	124,747.76	30,257.43	22,932.00	5205.56	476.76
Orthopedics	179,772.57	106,493.30	34,398.00	14,171.98	1396.64
Surgical wards TOTAL	804,439.01	465,461.76	150,969.00	69,594.80	841.53

**Table 3:** The cost-effect ratio concerning nurses’ higher education.

Wards	Number of Additional Hours of BSN/MSc Nurses (10%)	Cost of Additional Graduate Nursing Hours (USD)	The Number of Readmissions That Are Preventable through a 10% Increase in BSN/MSc Nurses	Cost-Effect Ratio with an Increase of 10% in the Hours of BSN/MSc Nurses (PLN)	Cost-Effect Ratio with an Increase of 10% in the Hours of BSN/MSc Nurses (USD)
Pulmonology	623.75	1907.94	6	1220.40	347.99
General Ward	2201.47	7225.54	19	1309.49	373.39
Neurology	1967.57	498.41	17	101.07	28.82
Cardiology	1109.91	2098.74	10	754.43	215.12
Non-surgical wards TOTAL	5902.70	11,730.62	52	792.90	226.09
General surgery	3678.68	5029.85	91	193.82	55.27
Urology	1343.05	1159.18	33	122.35	34.89
Laryngology	520.56	193.97	13	52.82	15.06
Orthopedics	1417.20	3242.70	35	324.35	92.49
Surgical wards TOTAL	6959.48	9462.55	172	192.74	54.96

**Table 4:** The parameters and calculations used in the sensitivity analysis.

	Parameters Used					
	Sensitivity Analysis Calculations in Non-Surgical Wards			Sensitivity Analysis Calculations in Non-Surgical Wards		
	Base Case	Upper Value	Lower Value	Base Case	Upper Value	Lower Value
The number of additional hours provided by nurses with high education	5902.70	6906.16	4899.24	6959.48	8490.57	5428.39
The cost of one nursing hour provided by a BSN/MSN nurse (PLN)	22.99	27.82	22.99 *	23.46	28.39	23.46 *
The cost of one nursing hour provided by a BSN/MSN nurse (USD)	6.56	7.93	6.56 *	6.69	8.09	6.69 *
The number of preventable readmissions	52	60	38	172	201	109

* no reduction in salaries was assumed.

**Table 5:** Sensitivity analysis.

*CER—Preventable Readmissions*						
	Increasing the Percentage of Hours of Care Provided by BSN/MSc Nurses by 10% in Non-Surgical Wards			Increasing the Percentage of Hours of Care Provided by BSN/MSc Nurses by 10% in Non-Surgical Wards		
	Variant			Variant		
	Standard	Pessimistic	Optimistic	Standard	Pessimistic	Optimistic
The number of nursing hours provided by BSN/MSc nurses (PLN)	792.9	990.93	702.47	192.74	235.14	150.34
The number of nursing hours provided by BSN/MSc nurses (USD)	226.09	282.56	200.31	54.96	67.05	42.87
The cost of one nursing hour provided by a BSN/MSc nurse (PLN)	792.9	1280.09	792.9	192.74	391.83	192.74
The cost of one nursing hour provided by a BSN/MSc nurse (USD)	226.09	365.01	226.09	54.96	111.73	54.96
The number of preventable readmissions (PLN)	792.9	944.54	617.85	192.74	197.68	182.69
The number of preventable readmissions (USD)	226.09	269.33	176.18	54.96	56.37	52.09

## Data Availability

The patients’ and nurses’ data cannot be shared by the investigators under the data use agreement with the hospital in Wejherowo; however, the original data collection can be requested directly from the hospital in Wejherowo.
